# Carbohydrates and secondary compounds of alpine tundra shrubs in relation to experimental warming

**DOI:** 10.1186/s12870-022-03851-y

**Published:** 2022-10-10

**Authors:** Yumei Zhou, Ming Yang, Zhijuan Tai, Jingjing Jia, Dongtao Luan, Xia Ma

**Affiliations:** 1 Ecological Technique and Engineering School, Shanghai Institute of Technology, Shanghai, 201418 China; 2 Department of Tourism Economy, Changbai Mountain Academy of Sciences, Baihe, 133633 China; 3 School of Perfume and Aroma Technology, Shanghai Institute of Technology, Shanghai, 201418 China

**Keywords:** Soluble sugars, Starch, Flavonoids, Phenols, Triterpenes, Coverage

## Abstract

**Background:**

It is critical to understand the sensitivity, response direction and magnitude of carbohydrates and secondary compounds to warming for predicting the structure and function of the tundra ecosystem towards future climate change.

**Results:**

Open-top chambers (OTCs) were used to passively increase air and soil temperatures on Changbai Mountain alpine tundra. After seven years’ continuous warming (+ 1.5 °C), the vegetation coverage, nonstructural carbohydrates (soluble sugars and starch) and secondary compounds (total phenols, flavonoids and triterpenes) of leaves and roots in three dominant dwarf shrubs, *Dryas octopetala* var. *asiatica*, *Rhododendron confertissimum* and *Vaccinium uliginosum*, were investigated during the growing season. Warming did not significantly affect the concentrations of carbohydrates but decreased total phenols for the three species. Carbohydrates and secondary compounds showed significantly seasonal pattern and species-specific variation. No significant trade-off or negative relationship between carbohydrates and secondary compounds was observed. Compared to *Dr. octopetala* var. *asiatica*, *V. uliginosum* allocated more carbon on secondary compounds. Warming significantly increased the coverage of *Dr. octopetala* var. *asiatica*, did not change it for *V. uliginosum* and decreased it for *Rh. confertissimum*. *Rh. confertissimum* had significantly lower carbohydrates and invested more carbon on secondary compounds than the other two species.

**Conclusions:**

Enhanced dominance and competitiveness of *Dr. octopetala* var. *asiatica* was companied by increased trend in carbohydrate concentrations and decreased ratio of secondary compounds to total carbon in the warming OTCs. We, therefore, predict that *Dr. octopetala* var. *asiatica* will continue to maintain dominant status, but the competition ability of *V. uliginosum* could gradually decrease with warming, leading to changes in species composition and community structure of the Changbai tundra ecosystem under future climate warming.

## Background

Global air temperature has been predicted to increase continuously, with the most marked increase in alpine and tundra regions [1]. During the past decades from 1950 to 2010, Changbai Mountain alpine tundra experienced significant increases in both temperature and precipitation and decreases in frost and ice days. The warming rate of growing season is 0.0239 °C y^-1^, higher than that of the average earth surface temperature. The ice day decreased 0.2245 d y^-1^ and the increased rate of potential evapotranspiration is 0.6312 mm y^-1^ [2]. In addition, it is hard to see the snow on the top of the Changbai Mountain in summer. Temperature change alone, and in combination with other environmental changes, will inevitably have considerable impacts on ecosystems [3]. Tundra vegetation on the Changbai Mountain has changed significantly over the last decades, showing that the abundance of shrubs decreased, whereas the grasses’ abundance continuously significantly increased [4]. For instance, the grasses that previously either occurred in the mountain birch forests at a lower elevation or were only occasionally observed in the tundra have extensively invaded the Changbai Mountain alpine shrub tundra, and the tundra vegetation is currently co-dominated by shrubs and six herb species (*Calamagrostis angustifolia*, *Geranium baishanense*, *Ligularia jamesii*, *Sanguisorba parviflora*, *S. stipulata*, and *Saussurea tomentosa*) [5, 6]. Jin et al. stated very recently that the Changbai Mountain shrub tundra will be replaced by a grass tundra, and which is mainly regarded as a consequence of continuous air warming [7].

Strong and sensitive reactions in phenology, photosynthesis, growth, leaf nutrients, and carbon contents and components of alpine and arctic plants to climate warming, including positive and negative responses, have been observed [8–12]. A question that remains unclear is whether species that respond negatively to warming will have disadvantages and thus must escape from the community under future climate warming. For example, it has been observed that two moss species completely disappeared, and the abundance of three dwarf shrub species decreased, but the abundance of forb and grass species did not change after four years’ warming in an alpine plant community, southwestern Norway [13]. Two previously dominant shrubs, *Rhododendron chrysanthum* and *Vaccinium uliginosum*, on the west-facing slope of Changbai Mountain alpine tundra showed different responses to climate change over the last decades. *V. uliginosum* significantly decreased its abundance and changed its previously even distribution pattern into a patch distribution, while *Rh. chrysanthum* did not [7]. Whether such decreased abundance of some species or disappearance of moss is related to physiologically negative responses to climate change, and/or whether the change in community structure can be explained by physiological competition relationships among species are poorly understood [13].

Competitive relationship among co-existing species is affected by many factors. The competitive ability of a species is influenced by available nonstructural carbohydrates (NSCs, mainly sugars and starch) and secondary metabolism and *vice versa* [14, 15]. Competition may affect the distribution and allocation pattern of carbon between growth and defense. NSCs and secondary compounds support plant physiological processes of vegetative and reproductive growth, maintenance, storage, and defense, hence, they may be allocated for various purposes, affecting competition or trade-offs between NSCs and secondary compounds at an individual level [16]. For example, more investment of carbon to plant growth could lower plant concentrations of carbon-based secondary compounds [17]. Growth on tundra is limited by low temperature. According to hypotheses of resource based defence, alpine plants might invest more carbon in defence. When temperature increases, increased resource availability will prioritize growth and spend less on defence [17]. The relative growth rate of four trembling aspen was negatively related to condensed tannin content [14]. The carbon allocation to defensive compounds decreased for tomato due to competition [18]. The competition led to a decrease in starch concentration for two *Larix* species, but soluble sugars and total NSCs (TNC) concentrations were not directly influenced by competition [15].

Changes in environmental conditions will alter the allocation of photosynthetic fixed carbon between primary and secondary compounds and the responses might be highly species specific. Aerts et al. observed that the carbon concentration of three co-existing sub-arctic dwarf shrubs – *Empetrum hermaphroditum*, *Andromeda polifolia* and *V. uliginosum* responded significantly differently to experimental warming [19]. Starch reserves increased across 14 tree species along a natural temperature gradient from lowland to the alpine treeline [20]. According to a simulated warming experiment, elevated temperature did not significantly change the concentrations of soluble sugars and starch in needles, bark and wood of *Larix decidua* grown at the alpine treeline [21]. The carbon-based secondary compound concentrations of *Tofieldia pusilla* were decreased by warming, while *Saussurea alpina*, *Carex vaginata*, *V. uliginosum*, *Selaginella selaginoides* in the same growth environment did not respond to the warming treatment [17]. The tannin concentrations of *Cassiope tetragona* and *V. vitis-idaea* leaves were increased by warming [22]. We wonder, therefore, whether a synchronized change or a trade-off between NSCs for growth and the secondary compounds for defense exists in tundra plants under global warming.

We used a 7-year experimentally warming site (+ 1.5 °C in the air temperature and + 0.8 °C in the soil temperature at 10 cm depth) in the alpine tundra on the north-facing slope of Changbai Mountain to study whether changes in plant coverage are associated with changes in carbon resource availability and allocation between growth and defense. We measured NSCs (soluble sugars, starch) and carbon-based secondary compounds (total phenols, flavonoids and triterpenes) in three dominated shrub species (*Dryas octopetala* var. *asiatica*, *V. uliginosum*, *Rh. confertissimum*) which have shown marked variation in their coverage responses to warming over time (see Table 1). We hypothesize that (1) a decrease in the coverage of species implies negative responses of NSCs to warming, and *vice versa*; (2) the levels of NSCs and carbon-based secondary compounds of the three species respond to warming differently, showing a significant interaction between species and warming; and (3) there is a trade-off between NSCs and secondary compounds to warming. We aimed to assess the effects of warming on NSCs and secondary compounds in dominant shrubs on alpine tundra, to investigate the link between the change in vegetation coverage and NSCs as well as secondary compounds, and thus to understand whether the plant carbon-physiological parameters can be used as useful indicators to predict vegetation change (winner or loser) under global warming or environmental changes.

**Table 1:** The average coverage of three shrub (*Dryas octopetala* var. *asiatica*, *Vaccinium uliginosum* and *Rhododendron confertissimum*) species in the warming open-top chambers and the control plots investigated before and after seven years’ warming treatment (unit: %; *n*=8). Different lowercase letters indicated significant difference in coverage among years. The statistical results of the effects of warming on coverage was shown by *P* value for each species (** *P* < 0.01, *** *P* ≤ 0.001, ns *P* > 0.05.)

	*Dr. octopetala* var. *asiatica*		*V. uliginosum*		*Rh. confertissimum*	
	Warming	Control	Warming	Control	Warming	Control
2010	38 ± 2.3 a	31 ± 3.7 a	29 ± 3.1 a	29 ± 4.3 a	9 ± 0.8 a	15 ± 2.0 b
2015	43 ± 5.8 a	31 ± 3.6 a	30 ± 2.8 a	28 ± 3.1 a	6 ± 0.9 b	19 ± 2.4 a
2016	33 ± 5.8 a	20 ± 2.1 b	33 ± 2.7 a	27 ± 3.6 a	< 5 b	12 ± 2.1b
2017	32 ± 6.2 a	24 ± 1.8 ab	33 ± 4.1 a	29 ± 3.2 a	< 5 b	14 ± 2.4 b
*P* value	**		ns		***	

## Results

### Responses of species coverage to warming

Experimental warming significantly increased the coverage of *Dr. octopetala* var. *asiatica* (*P* < 0.01), but it highly significantly decreased (*P* < 0.001) the coverage of *Rh. confertissimum* (Table 1). The coverage of *V. uliginosum* was not markedly changed by warming (Table 1). After seven years of warming treatment, the coverage of *Rh. confertissimum* in the warming OTCs was very small, less than 5% (Table 1).

### Leaf nonstructural carbohydrates

**Fig. 1:**
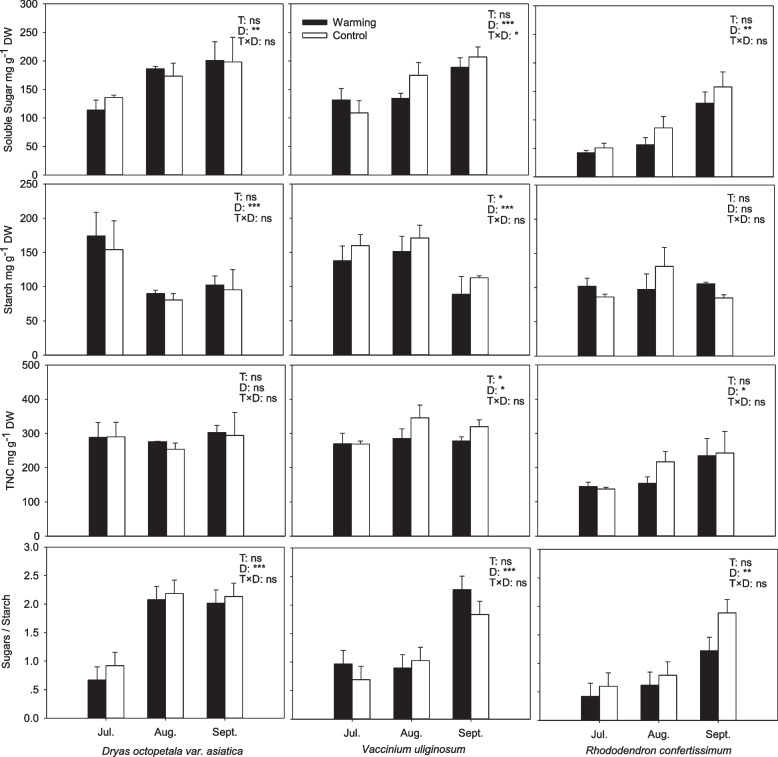
Concentrations of soluble sugars, starch, TNC (sugar + starch) and the ratio of sugars to starch in leaves of *Dryas octopetala* var. *asiatica*, *Vaccinium uliginosum* and *Rhododendron confertissimum* grown in the warming open-top chambers (black bars) and the control plots (white bars) measured during the growing season. Error bars are one standard deviation (*n* = 6). The effects of treatment (T) and sampling date (D) on all parameters were analyzed by two-way ANOVA. * *P* < 0.05, ** *P* < 0.01, *** *P* ≤ 0.001, ns *P* > 0.05

Warming treatment had no marked effects on leaf soluble sugars, starch and TNC concentrations, as well as the ratio of sugars to starch, but all the four traits varied over time and were significantly species-specific (Table 2). Generally, the concentrations of soluble sugars were relatively higher, and the starch concentration was lower in both *Dr. octopetala* var. *asiatica* and *V. uliginosum* (Fig. 1).

**Table 2:** The statistical results of the effects of treatment (warming and control), sampling dates (July, August, September) and species (*Dryas octopetala* var. *asiatica*, *Rhododendron confertissimum* and *Vaccinium uliginosum*) on the soluble sugars, starch and total nonstructural carbohydrate (TNC) (sugar + starch) concentrations, the ratio of soluble sugars to starch, and secondary compounds (flavonoids, total phenols, triterpene) concentrations in leaves using three-way ANOVA

	Sugar		Starch		TNC		Sugar/Starch		Flavonoids		Total phenols		Triterpene	
	F	*P*	F	*P*	F	*P*	F	*P*	F	*P*	F	*P*	F	*P*
Treatment (T)	2.448	ns	0.164	ns	1.982	ns	0.851	ns	1.790	ns	229.742	***	0.021	ns
Sampling dates (D)	38.712	***	8.798	***	6.042	**	38.590	***	1.070	ns	10.723	***	72.249	***
Species (S)	43.192	***	7.976	***	39.783	***	15.228	***	22.136	***	359.343	***	775.940	***
T × D	0.399	ns	0.644	ns	0.916	ns	0.055	ns	28.124	***	250.941	***	171.625	***
T × S	0.553	ns	1.877	ns	1.465	ns	2.046	ns	3.416	ns	16.275	***	73.310	***
D × S	1.345	ns	7.691	***	3.278	ns	5.164	**	0.724	ns	266.306	***	180.204	***
T × D × S	1.171	ns	0.658	ns	0.741	ns	0.758	ns	5.466	**	202.599	***	75.168	***

Notes: Significance ** *P* < 0.01, *** *P* ≤ 0.001, ns *P* > 0.05

Starch and TNC concentrations of *Dr. octopetala* var. *asiatica* in the warming OTCs were 11% and 4% higher than those in the control across the whole growing season although the difference was not statistically significant. The soluble sugars of *Rh. confertissimum* in the warming decreased by 17% in July, 34% in August and 18% in September compared to the control, but starch concentration did not change with warming across the whole growing season (Fig. 1). We observed that warming decreased starch concentration of *V. uliginosum* leaves by 14% in July, 12% in August and 21% in September, on average 15% decline across the whole growing season (*P* = 0.003). Therefore, the ratio of sugars to starch of *V. uliginosum* leaves in the warming was higher than that in the control, but the difference was not significant (Fig. 1).

Soluble sugars, starch and TNC in leaves of the three species showed a pronounced seasonal variation for both the warming OTCs and the controls. Soluble sugars and the ratio of sugars to starch increased with time, reaching the maximum values for the three species in September when the temperature was relatively lower (Fig. 1). The starch concentrations of *Rh. confertissimum* in the warming OTCs were relatively stable during the whole growing season (Fig. 1). The seasonal trend of starch in *Dr. octopetala* var. *asiatica* and *V. uliginosum* showed an opposite trend of soluble sugars, and therefore, the TNC was relatively stable with time. However, the TNC concentrations of *Rh. confertissimum* leaves increased with time, reflecting the seasonal pattern of soluble sugars (Fig. 1).

The concentrations of soluble sugars and TNC did not differ between *Dr. octopetala* var. *asiatica* and *V. uliginosum* (*P* > 0.05) but significantly higher than *Rh. confertissimum* (*P* = 0.007). The soluble sugars concentrations of *Dr. octopetala* var. *asiatica* and *V. uliginosum* were approx. 1.9 times that of *Rh. confertissimum*. There was no significant difference in starch concentration among the three species, ranging from 101.2 to 136.9 mg g^-1^ when all data were pooled. The TNC concentration had a similar trend to soluble sugars (Fig. 1). The TNC concentration of *Dr. octopetala* var. *asiatica* and *V. uliginosum* was approx. 1.5 times that of *Rh. confertissimum*.

### Carbon-based secondary compounds in leaves

Warming significantly affected the concentrations of total phenols (*P* < 0.001) and flavonoids of *Rh. confertissimum* (*P* = 0.026). Warming decreased the total phenols concentrations by 16% for *Rh. confertissimum*, 21% for *Dr. octopetala* var. *asiatica* and 7% for *V. uliginosum* across the growing season. On average, *Rh. confertissimum* in the warming OTCs had 14% higher flavonoids concentration than the controls across the whole growing season.

**Fig. 2:**
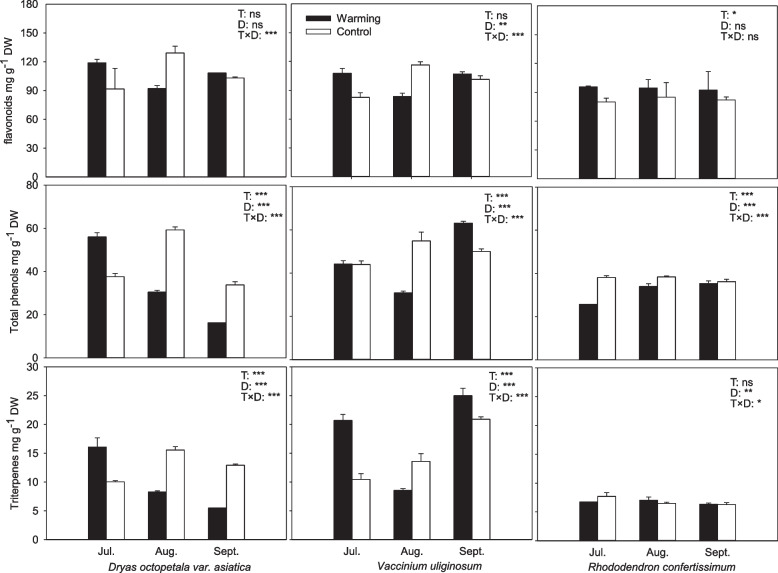
Concentrations of flavonoids, total phenol and triterpenes in leaves of *Dryas octopetala* var. *asiatica*, *Rhododendron confertissimum* and *Vaccinium uliginosum* grown in the warming open-top chambers (black bars) and the control plots (white bars) measured during the growing season. Error bars are one standard deviation (*n* = 6). The effects of treatment (T) and sampling date (D) on all parameters were analyzed by two-way ANOVA. * *P* < 0.05, ** *P* < 0.01, *** *P* ≤ 0.001, ns *P* > 0.05

Total phenols and triterpenes showed pronounced seasonal fluctuation, but different species had different patterns (Fig. 2). Total phenols and triterpenes of *Dr. octopetala* var. *asiatica* grown in the warming OTCs decreased with time, but the controls had the highest values in August. *V. uliginosum* had relatively higher total phenols and triterpenes in September. The flavonoids were stable during the growing season for the three species.

There were significant differences in flavonoids, total phenols and triterpenes concentrations among the three species (Table 2). *Rh. confertissimum* had the lowest concentrations of flavonoids, total phenols and triterpenes compared to *Dr. octopetala* var. *asiatica* and *V. uliginosum* (Fig. 2). The total phenols concentration of *V. uliginosum* were 1.4 times higher than *Dr. octopetala* var. *asiatic*from and *Rh. confertissimum* in the warming and approx. 1.2 times higher in the controls. *V. uliginosum* had 1.4 and 2.4 times higher triterpenes concentration than *Dr. octopetala* var. *asiatic*from and *Rh. confertissimum* across the whole growing season, with the highest difference mainly occurring in September (Table 2, Fig. 2).

### Nonstructural carbohydrates in roots

**Fig. 3:**
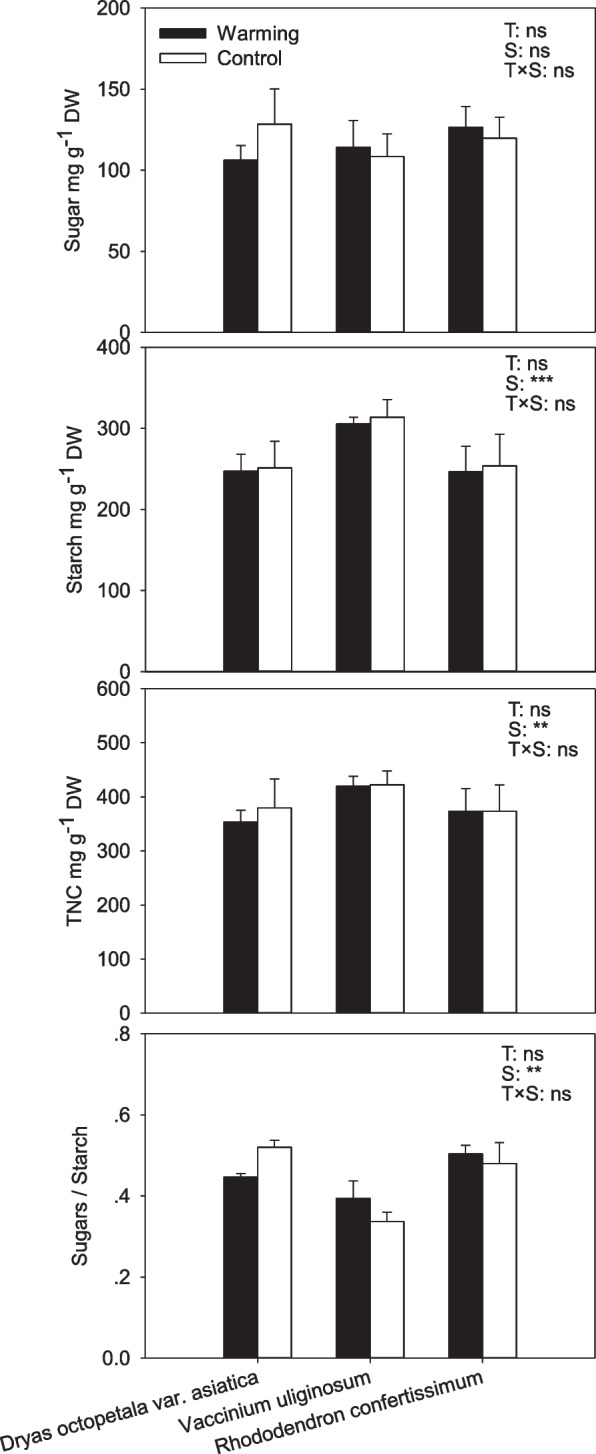
Soluble sugars, starch, total carbohydrate concentrations (TNC) and the ratio of sugars to starch in roots of *Dryas octopetala* var. *asiatica*, *Vaccinium uliginosum* and *Rhododendron confertissimum* from the warming open-top chambers (black bars) and the controls (white bars) measured at the end of the growing season. (mg g^−1^ ± SD) (*n* = 6). The effects of treatment (T) and species (S) on all parameters were analyzed by two-way ANOVA. ** *P* < 0.01, *** *P* ≤ 0.001, ns *P* > 0.05

No significant effects of warming on soluble sugars, starch and TNC concentrations and the ratio of sugars to starch in roots were observed for the three species, but there were significant differences among species (Fig. 3). *V. uliginosum* roots had 24% higher starch concentration and 12% higher TNC concentration compared to *Dr. octopetala* var. *asiatica* and *Rh. confertissimum*. The TNC concentration in *Dr. octopetala* var. *asiatica* roots was very close to *Rh. confertissimum* (Fig. 3).

## Discussion

### Warming effects on carbohydrates and their possible effects on coverage

Experimental warming in tundra regions often causes increased photosynthesis and growth rate [23–25]. In line with our hypothesis 1, *Dr. octopetala* var. *asiatica* positively responded to OTC-warming in tissue NSCs, plant coverage, photosynthesis and single leaf size [25], indicating that warming makes *Dr. octopetala* var. *asiatica* to fix more carbon, to grow fast, and then to occupy more space and have stronger competitiveness. The soluble sugars of *Rh. confertissimum* negatively responded to warming (Fig. 1), and decreases in the coverage of *Rh. confertissimum* have already been recorded during the experimental period (Table 1), consistent with the hypothesis 1. The coverage of *V. uliginosum* grown in the warming OTCs and the control plots did not significantly change during the whole experimental period (Table 1). Jin et al. (2019) found that *V. uliginosum* on the west-facing slope of Changbai Mountain alpine tundra showed a patch distribution deviating from the previously normal distribution [7]. Our research field was located on the north-facing slope whose microenvironment (e.g. lower temperature) should be different from the west-facing slope (higher temperature) of Changbai Mountain alpine tundra. The starch storage of *V. uliginosum* responded negatively to the warming (Fig. 1), so we predict that the distribution of *V. uliginosum* on the north-facing slope might decrease in the future or gradually show a patch distribution like on the west-facing slope.

The responses of carbohydrates to warming in tundra shrubs were found to be species-specific in the present study which is consistent with our hypothesis 2 (Table 2). Similar results were also observed by other studies [26, 27]. Warming increased NSCs concentrations in *Himantormia lugubris* but decreased them in *Polytrichastrum alpinum*, *Pinus sylvestris*, *Pseudotsuga menziesii* and *Picea mariana* [27–30]. No significant change in TNC was observed in *Salix Polaris*, *Carex vaginata*, *Saussurea alpine*, *Selaginella selaginoides*, *V. uligonosum*, *Usnea antarctica*, *U. aurantiaco-atra*, *Sanionia uncinata*, *Quercus robur* and *Q. petraea* leaves in response to warming [17, 24, 26, 27]. Different species have different sensitivity to warming which can affect photosynthesis, the distribution and utilization of photosynthate, and growth etc.

Stored carbohydrates in roots can be used by plants for defense and regrowth, or as a buffer under insufficient carbon production [31–35]. Warming did not significantly affect root carbohydrate concentrations of the three species, which is consistent with previous findings that there were non-significant responses of root carbohydrates at the end of the growing season to warming for alpine plants (*Elymus nutans*, *Euphrasia regelii* and *Swertia mussotii*) on the Tibetan Plateau [10], for grapevines in Barossa Valley of Australia [33], for *Pinus taeda* and *P. ponderosa* [36]. *V. uliginosum* roots had higher starch and TNC concentrations compared to *Dr. octopetala* var. *asiatica* and *Rh. confertissimum,* which might be related to that *V. uliginosum* is a deciduous species. Deciduous plants need more energy and nutrition for new leaves sprouting in the next spring, especially on alpine tundra regions.

The accumulation of NSCs is one of the cryoprotective mechanisms [37]. The sugar-starch system in plants adjusts the ratio of sugar to starch in response to low temperature or other stressors [38]. At high elevations, a higher sugar-starch ratio reflects that plants are subjected to lower temperatures, sometimes positively correlating with cold stress [39]. We found that the ratios of soluble sugars to starch in the warming OTCs were lower than those in the control plots for all the three species, indicating that warming can affect sugar-starch relationship. The three species grown in the warming OTCs hydrolyze less starch against freezing which might be beneficial to growth.

### Time-dependent NSC levels and their possible effects on coverage

Marked seasonal patterns of NSCs concentrations for the three species were observed, which is in agreement with earlier findings of the seasonal NSCs fluctuation with soluble sugars concentrations reaching a higher level at the end of the growing season in various tree species [40–42]. Soluble sugars, serving as osmotic adjustment and signal substances, play an important role against cold [41, 43]. The starch concentrations peaked in July or August (the active growing season) and decreased in September, indicating that starch hydrolyzes to soluble sugars towards end-season [42]. The temporal variation in the level of NSCs also illustrates that temperature might affect the proportions of carbohydrate component.

Wintergreen *Dr. octopetala* var. *asiatica* and deciduous *V. uliginosum* are typical alpine and arctic dwarf shrubs, especially *Dr. octopetala* var. *asiatica* generally dominating community [44]. Evergreen *Rh. confertissimum* has a small distribution area compared to the two others, only in tundra regions. Generally, deciduous trees require more abundant carbohydrates for vegetative or reproductive growth before the new leaves grow [34, 45]. *V. uliginosum* had similar concentrations of TNC as *Dr. octopetala* var. *asiatica*, but *V. uliginosum* did not show the same obvious advantages in coverage, photosynthesis, leaf size and growth as *Dr. octopetala* var. *asiatica* did [25], indicating that *V. uliginosum* might allocate some carbohydrates to the growth of new organs. The roots of *V. uliginosum* had significantly higher starch and TNC concentrations than *Dr. octopetala* var. *asiatica* and *Rh. confertissimum*. Starch, different from soluble sugars, is inactive and accumulates as a storage compound. *V. uliginosum*, as a deciduous species, is assumed to store high amounts of carbohydrates over harsh winter to support leaf flush in spring [45]. The concentrations of TNC in *V. uliginosum* and *Dr. octopetala* var. *asiatica* were significantly higher than those of *Rh. confertissimum* that had significantly decreased coverage. Therefore, TNC concentrations are related to the species coverage, which is consistent with our hypothesis 1.

The NSCs responses of *Dr. octopetala* var. *asiatica* to warming support the idea that carbon allocation is a key factor for determining dominance. After five years of warming by open-top chambers in the alpine region of southwestern Norway, the carbohydrates storage of *Dr. octopetala* increased [46]. *Dr. octopetala* var. *asiatica* showed increased trend in the carbohydrate concentrations in the present study and significantly increased leaf size [25], indicating that the total carbohydrate contents (leaf biomass or size × concentration) have been stimulated. The stimulation and accumulation of carbohydrates are conducive to dominance, expansion and improvement of competitiveness, which may further lead to changes in community composition and structure of alpine and tundra ecosystems under future climate change [44].

### Responses of secondary compounds and trade-off with NSCs

Deciduous species probably have lower concentrations of secondary compounds compared to evergreen plants [47]. Higher concentrations of secondary compounds in perennial leaves could be a greater need for defending herbivorous predator due to longer life span [48]. However, we found that the deciduous *V. uliginosum* had the highest absolute concentrations of total secondary compounds compared to the other two species. One of the reasons is probably related to that *V. uliginosum* produces delicious fruits which might need more secondary compounds to defend animals, especially triterpenes.

We observed that *Rh. confertissimum* allocated relatively more carbon to defense than *V. uliginosum* and *Dr. octopetala* var. *asiatica* based on the ratio of the sum of secondary compounds to total carbon (secondary compounds + TNC). Herms and Mattson (1992) found that increased investment in secondary defense is accompanied by decreased growth, plant size and competitive ability [16]. Thus, more carbon is allocated to secondary compounds in expense of growth, dominance and competition. Warming may alter interspecific competitive relationships and community structure because of the changes in carbon allocation pattern and defense abilities [46].

Total phenols concentrations were decreased by warming in the present study, similar to the results of Holopainen et al. [49]. Phenolic compounds originate from the shikimic acid pathway which is related to the carbohydrate metabolisms [50, 51] and antioxidative potential of plants [52]. Tundra ecosystem is generally characterized by simultaneous stresses such as low temperature, high UV radiation, low nutrient availability which could make plants to produce high levels of secondary compounds or allocate more proportion carbon to the secondary compounds [46, 53]. Thus, alleviation of low temperature by warming is expected to decrease the contents of secondary compounds. The decreases in the concentrations of secondary compounds for *Bistorta vivipara*, *Dr. octopetala*, *Salix reticulate*, *Cassiope tetragona*, *S. herbacea* × *Polaris* and *Tofieldia pusilla* have been reported [17, 22, 46]. The decrease in total phenols concentration probably relates to the fewer carbon resources for defense substance or more carbon for growth [17]. The decreased proportion of total phenols of *Dr. octopetala* var. *asiatica* was relatively higher than the other two species (Fig. 2) which is consistent with higher NSCs and growth. Hence, *Dr. octopetala* var. *asiatica* has strong competitive potential.

Flavonoids concentrations of *Rh. confertissimum* were increased by warming, but OTC warming did not affect the levels of flavonoids in *Dr. octopetala* var. *asiatica* and *V. uliginosum*. We also found that *Rh. confertissimum* grown in the warming OTCs had relatively lower soluble sugars and significantly reduced coverage than the controls or the other two species. The three species in the present study changed their defense levels to some extent when experiencing continuous 7 years’ warming, consistent with our hypothesis 2 that there was significant interaction between species and warming on the total phenols and flavonoids.

Inconsistent with our hypothesis 3, no significant trade-off relationship between NSCs and secondary compounds in leaves was observed based on the correlation analysis (Figure not shown). However, the secondary compounds tended to be positively correlated with NSCs for *Dr. octopetala* var. *asiatica* while negatively correlated with NSCs for *V. uliginosum* and *Rh. confertissimum*. Further research is necessary to continue to examine if long-term warming can result in a trade-off relationship between NSCs and secondary compounds for species with decreased distribution or competitiveness.

## Conclusions

We conclude that warming increased NSCs and decreased secondary compound in *Dr. octopetala* var. *asiatica*, which makes the species still maintain dominant status in the Changbai Mountain alpine tundra with climate change. The dominance of *V. uliginosum* could gradually decline with continuous warming. *Rh. confertissimum* had relatively lower carbohydrates compared to *Dr. octopetala* var. *asiatica* and *V. uliginosum* and increased secondary compounds investment with warming. The coverage of *Rh. confertissimum* in the OTCs was getting small. All these traits will weaken the competition ability of *Rh. confertissimum* in tundra community. Thus, we predict that *Dr. octopetala* var. *asiatica* will maintain its dominant status, *V. uliginosum* could gradually decrease its coverage, and *Rh. confertissimum* might be in danger of disappearance with air warming, leading to changes in species composition and community structure of the Changbai tundra ecosystem under future climate warming.

## Materials and Methods

### Study site and experimental design

The study was conducted on the north-facing slope of alpine tundra on Changbai Mountain (41°58´- 42°42´N; 127°67´- 128°27´E, 2046 m a.s.l.), northeastern China with typical characteristics of Arctic tundra [54]. Changbai Mountain alpine tundra experienced significant increases in temperature and precipitation and decreases in frost and icing days based on the data from 1950 to 2010 [2]. The mean annual temperature is -7.3 °C and mean annual precipitation is 1373 mm in this region [55]. The mean air temperature during the growing season (June to September) is 5.9 °C, and the highest mean daily temperature is less than 10 °C [2]. The majority falling as rain occurs during the short summer (July and August). A snow-free season lasts from May to September. Soils are characterized by Haplic Cambisol (Humic, Dystric). The total nitrogen, phosphorus and potassium were 3.6 ± 0.19, 1.00 ± 0.06 and 1.64 ± 0.01 mg/g, respectively, and the soil had an organic matter content of 124 ± 9.2 mg/g. The vegetation mainly consisted of *Dr. octopetala* var. *asiatica*, *V. uliginosum*, *Rh. confertissimum*, *Rh. chrysanthum*, *Sanguisorba parviflora*, *S. stipulate*, *Calamagrostis angustifolia*, etc.

Open-top chambers (OTCs) were used to increase air and soil temperature according to the criteria of the International Tundra Experiment [56]. Eight OTCs were established near the viewing platform in June 2010, about half a mile away from road, with a steep slope in the middle. The OTCs were hexagonal, 0.45 m high, had inclined sides (60°), enclosed a surface of 1.0 m^2^, and were made of transparent polycarbonate. The OTCs were left in place year-round. Control plots were established beside each OTC with similar species composition and vegetation coverage. Air and soil temperature, air and soil humidity, radiation in the OTCs and the control plots were logged every 30 minutes during growing seasons (June to September) by Em 50 Data Collection System (Decagon, USA). On average, the OTC increased air and soil temperature at 10 cm depth by 1.5 °C and 0.8 °C (with less increase in air temperature, while decrease in soil temperature during night). The air relative humidity was not significantly changed by warming, but the soil water content was decreased by 0.05 m^3^ m^-3^ [25].

Three dominant species growing on Changbai Mountain tundra were selected in the present study. *Dr. octopetala* var. *asiatica* is an evergreen dwarf shrub and often forms heath communities on calcareous soils in arctic-alpine environments [46]. *V. uliginosum* is deciduous dwarf shrub with thin and approximate round leaves. *Rh. confertissimum* is an evergreen dwarf shrub with relatively thicker leaves. The specimens of the three plants can be found in the Institute of Applied Ecology, Chinese Academy of Sciences with voucher ID C.Y. Li 1962, Y.L. Zhou 1951, and P.Y. Fu 1959.

### Sampling leaves and roots

Experimental area and sampling for scientific research were approved by local administrative department that supported the present project. We identified the three plant species. After all samples were dried and ground in the field station, they were stored in the laboratory in Shanghai Institute of Technology.

The leaves of *Dr. octopetala* var. *asiatica*, *V. uliginosum*, *Rh. confertissimum* were sampled from three plants in each OTC and the control plot on July 13th, August 13th and September 18th, 2017. The roots were only sampled on September 18th because of the destructive sampling. Plants were always randomly sampled away from the chamber sides.

### Nonstructural carbohydrates analysis

Dried leaves and roots were ground into fine powders using a small grinder. Extraction and determination of carbohydrates from leaves and roots were based on the Anthrone method [57]. The powdered material was suspended in 80% ethanol and incubated for 30 min at 80°C for extraction of soluble sugars. The supernatant was decanted after centrifugation. Then the residual was resuspended in 80% ethanol and repeated the same procedure twice. The supernatant was in constant volume and then was quantified using anthrone as a reagent at 620 nm. The remaining pellet was kept for starch analysis. The pellet was washed two times with 4.6 mol/L HClO_4_ to digest starch. The supernatant was quantified using the same method as soluble sugars. TNC were calculated by summing total soluble sugars and starch [58].

### Secondary compounds analysis

Due to an insufficient number of root samples, only leaf secondary compounds were measured. Secondary compounds of total phenols, flavonoids and triterpenes were extracted by adding air dried leaf powder to 70% ethanol that was heated to reflux for two hours. The procedure was repeated two times. The filtered extract was condensed with a vacuum evaporator. Total phenols were measured at 760 nm wavelength by the Folinol method [59]. Flavonoids were determined at 510 nm wavelength with Rutin as the standard solution [60]. Triterpenes were measured at 542 nm wavelength with vanillin and glacial acetic acid as the chromogenic reagent [61].

### Data analysis

Twenty-four plants were respectively selected in the warming OTCs and the control for each species. The leaves of *Rh. confertissimum* were not enough to measure all parameters, so every four plants were mixed into one sample. MANOVA was used to test the significance of main effect factors (warming treatment, species and sampling date) and their interactions on carbohydrates and secondary compounds. Due to the overall significant effects of species on parameters studied, repeated measures ANOVAs were used to analyze the effects of treatment (between subject), sampling date (within subject), and their interaction on parameters within each species. Multiple comparisons were used to examine the difference in levels of the parameters among the three species. Paired-samples T test was used to assess the effects of warming treatment on every parameter for each species for each sampling date. Data were analyzed statistically using SPSS 16.0 system (SPSS Inc., Chicago, IL, USA). All tests of statistical significance were conducted at a level of 0.05.

## Data Availability

The datasets used and analyzed during the current study are available from the corresponding author on reasonable request.

## Abbreviations

OTCs: Open-top chambers
NSCs: non-structural carbohydrates
TNC: total NSCs
